# Faculty and resident perspectives on ambulatory care education: A collective case study of family medicine, psychiatry, and surgery

**Published:** 2017-06-30

**Authors:** Paula Veinot, William Lin, Nicole Woods, Stella Ng

**Affiliations:** 1 Centre for Ambulatory Care Education, Women’s College Hospital, University of Toronto, Ontario, Canada; 2 The Wilson Centre, University of Toronto, Ontario, Canada; 3 Department of Family and Community Medicine, University of Toronto, Ontario, Canada; 4 Centre for Faculty Development, University of Toronto, Ontario, Canada; 5 Department of Speech-Language Pathology, University of Toronto, Ontario, Canada

## Abstract

**Background:**

Ambulatory care (AC) experiences within medical education are garnering increasing attention. We sought to understand how faculty and residents’ describe their experiences of AC and ambulatory care education (ACEduc) within, between, and across disciplinary contexts.

**Methods:**

We designed a Stakian collective case study, applying constructivist grounded theory analytic methods. Using purposive and snowball sampling, we interviewed 17 faculty and residents across three instrumental cases: family medicine, psychiatry, surgery. Through constant comparative analysis, we identified patterns within, between, and across cases.

**Results:**

Family medicine and psychiatry saw AC as an inherent part of continuous, longitudinal care; surgery equated AC with episodic experiences in clinic, differentiating it from operating. Across cases, faculty and residents cautiously valued ACEduc, and in particular, considered it important to develop non-medical expert competencies (e.g., communication). However, surgery residents described AC and ACEduc as less interesting and a lower priority than operating. Educational structures mediated these views.

**Conclusion:**

Differences between cases highlight a need for further study, as universal assumptions about ACEduc’s purposes and approaches may need to be tempered by situated, contextually-rich perspectives. How disciplinary culture, program structure, and systemic structure influence ACEduc warrant further consideration as does the educational potential for explicitly framing learners’ perspectives.

## Introduction

Ambulatory care (AC) occurs in community-based primary care and outpatient settings, instead of inpatient, hospital-based environments,^1^ and, in part, aims to address budgetary and resource concerns.^2^ In contrast to the healthcare system’s growing reliance on AC,^1^ most medical training still relies on inpatient settings.^3^ Postgraduate trainees, often called residents, may lack confidence and real-world experience in treating patients with outpatient clinical problems,^4^ as the specialized and acute care focus of the inpatient context can divert educational attention away from common, chronic medical conditions.^5^–^7^ As healthcare systems shift focus towards higher quality outpatient care, medical education must keep pace. Learners need to experience ambulatory care education (ACEduc) so that they are prepared to care for patients outside of a hospital setting.

While calls for increased emphasis on AC and ACEduc have a long history,^8^ they remain timely and relevant.^9^–^12^ Twenty years ago, Schroeder et al.^8^ and Perkoff^9^ described challenges similar to those still faced today, including a changing case mix in academic hospitals, new responsibilities for staff due to changing compensation models, increased expectations for patient-centred and community-based medicine, and a growing need for primary care physicians. Similarly, the solutions proposed twenty years ago match those described in more recent literature, namely reforms to both medical education and physician practice models.^3^,^8^,^9^,^13^,^14^ The persistence of these problems and the reappearance of suggestions to address them speaks to the difficulty of system reform.

Encouraging, examples of ACEduc implementation with positive outcomes can be found in the recent literature.^3^ Numerous ACEduc programs have been implemented and evaluated, including pilot clinics, formal block rotations in medical school curricula, and academic institutions dedicated to ACEduc.^6^,^15^–^18^ ACEduc offers an opportunity to develop non-medical expert (sometimes referred to as “intrinsic”) roles^19^,^20^ like communication skills, health advocacy, and outpatient management,^3^,^21^,^22^ and has been shown to meet these educational objectives.^23^–^28^ Fiddes et al.^28^ describe ACEduc as a platform for patient-centered care, interprofessional education, and real-world reflection on ethics, professional relationships, and values.

Progress in AC and ACEduc continues, but confusion remains about the distinct purpose and definition of ACEduc, particularly from the perspectives of both learners and teachers. Medical education requires empirical, learner and teacher understandings of the particularities and contingencies of what AC or ACEduc are, and what makes them work well, the relationships between AC and ACEduc, and the relationships between ACEduc and the broader healthcare and education systems. Educationally, perspectives matter because how learners and teachers conceptualize AC and ACEduc speaks to how such learning experiences are framed and structured for learners, and may point to opportunities to reframe and re-structure. More broadly, an understanding of what ACEduc really looks like to those experiencing it matters for the reason that disconnects between AC, ACEduc, societal needs, and health systems likely affects patient care. Therefore, we asked a question to begin filling this gap: within situated residency program contexts, what are faculty and resident perspectives on AC and ACEduc, and how (if at all) do they differ between or align across these programs?

## Methods

### Research design

We designed a Stakian qualitative collective case study comprised of instrumental cases, wherein the cases were defined as three purposively chosen local residency programs. Instrumental case study, as a research methodology, selects a particular context with particular boundaries (in this study, discipline-specific residency programs, e.g. family medicine) as a case, and then offers important insight into the broader phenomenon represented through that case. This insight is situated within, but not focused upon, the case itself;^29^–^31^ case studies are thus useful when the phenomenon of interest is inseparable from its context.^32^–^37^ The methodology of collective case study combines multiple instrumental case studies, chosen to be similar and/or dissimilar, which together show what might be common across, and different between, cases of the same phenomenon, thus supporting theoretical explanation of the phenomenon being studied.^37^ As case study methodology does not itself specify data collection and analysis methods, we borrowed^38^ from the theoretically-aligned – and commonly paired with case study – data analysis methods of constructivist grounded theory.^39^–^41^

### Sampling

To construct the collective case, we sampled three instrumental cases, with each case bounded by its residency program. We selected our local residency programs for family medicine, psychiatry, and surgery. These cases were purposively sampled for their potential to reveal commonality and difference (or contingencies) in AC and ACEduc experiences.^36^,^42^–^45^ Therefore, based on our own understanding, we chose one case in which learners have greater exposure to AC and ACEduc (family medicine), one that offers a fairly even mix (psychiatry), and one for which AC and ACEduc comprise less time given the centrality of inpatient care (surgery). Our local AC and ACEduc contexts may not exactly mirror those of other contexts across Canada and the world; however, case study as a methodology specifically uses the thorough description of *one instance* of a common phenomenon to shed light on that phenomenon in the context of the case. Of course, a medical school in another province, country, or continent may have different factors to consider but, the contextual description derived through the case study methodology allows for local factors to be considered.

Within each case, we sampled for sufficient case descriptions, thus drawing from faculty and resident interview and sampling for curricular documents routinely provided to residents to orient them to AC or ACEduc. Within-case sample size, as in this research design, does not aim for “saturation” of findings *per se*, but rather for sufficiency of information power in relation to the instrumental case, and the research question.^39^,^45^–^47^ Information power is determined by the following: aims of the study, sample specificity, use of established theory, quality of dialogue, and analysis strategy.^48^ The aims of this study were to richly describe case-situated experiences. Given the specificity of the samples within cases, this aim for rich description was achieved after a small number of interviews for each case. Theoretical sensitivity did not explicitly inform data analysis since the study aimed for description and not theory-building. However, existing literature did guide study design and interpretation of findings for discussion. Rich interview dialogue was garnered by an experienced interviewer though the aim was not to generalize between or beyond the study’s cases. While the collective case study design did afford comparison of sorts across cases, it was not in a way that would presume that, for example, this study’s case of family medicine represents all instances of AC and ACEduc in family medicine. Such would be an incorrect assumption for interpretive qualitative research. Rather, the aim was to descriptively depict contextually-situated experiences of AC and ACEduc and provide some insights into the similarities and differences between and among the different programs.

### Recruitment and data collection

Interested residents responded to mass emails or group announcements distributed by PV, the study’s interviewer. The research team identified potential faculty participants and PV invited them by email. Participants were also asked to recommend our study to others whom they deemed suitable (snowball sampling).^42^ Prior to conducting the interviews, the interviewer obtained written consent, complying with the University of Toronto Research Ethics Board-approved protocol for the study. Interviews were audio-recorded and transcribed verbatim for analysis. PV documented reflexive field notes following each interview. The semi-structured interviews focused on AC and ACEduc understandings and experiences, perceived ACEduc learning opportunities, time spent in AC versus inpatient care, ACEduc content and structure, potential for competency development in ACEduc, and any other pertinent insights.

### Data analysis

All data (transcripts, field notes, curricular documents) were entered into Dedoose^48^ for data management. While program documents could have provided a contextualizing lens for analysis, the poor return of documents from the sites minimized this opportunity. We used constant comparison to analyze transcripts.^39^,^40^ Analysis involved initial coding (by PV, WL), focused coding to group similar codes together as categories and then, informed by authorship team discussions and reading of the reflexive field notes, theoretical coding to identify relationships between categories and to relate findings to extant literature.^39^ This process, concurrent with sampling and data collection, took place to the point of sufficient information power in relation to the research question, in accordance with the methodology’s standards.^39^,^45^–^47^ Our collective case study design led us to conduct this analytical approach within-, between-, and across-cases.

## Results

### Instrumental case descriptions

We were able to garner sufficient information power to achieve rich and consistent within-case descriptions after 17 interviews were conducted and analyzed. Buoyed by the within-case richness and consistency, between-case differences and across-case similarities became apparent with this sample. The collective sample of seven faculty and ten residents broke down as follows: family medicine (2 faculty, 4 residents), psychiatry (2 faculty, 2 residents), and surgery (3 faculty, 4 residents, from general surgery, orthopedics, plastic surgery).

The family medicine case was a two-year residency program with a number of core rotations (e.g., Internal Medicine, Surgery, Mental Health), and a two-month block in a rural teaching practice. The psychiatry case was a five-year program with core rotations in adult, child and geriatric psychiatry. The surgery case was a five-year program with mandatory and elective rotations (e.g., rural surgery). All programs can lead to sub-specialty opportunities. Residents rotate through hospitals to receive an adequate cross-section of training. All programs offered opportunities in core academic and affiliated community hospitals through block-learning formats and formal classroom teaching on academic half days. Faculty participants represented a range of clinical/teaching experience (2.5–21 years in practice) and residents spanned different stages of training (postgraduate years 1 through 6). Interviews lasted 34–65 (mean=54; median=56) minutes for residents and 35–65 (mean=48; median=46) minutes for faculty.

### Within-, between-, and across-case findings

Table 1 provides a summary of within-, between-, and across-case findings. To be concise, the written findings focus on between- and across-case findings, which inherently illuminate within-case findings. To provide context for the representative quotes we share, “F” refers to faculty and “R” to residents, “FM” refers to family medicine, “P” to Psychiatry, and “S” to Surgery. The numbers following these designations indicate when in the sequence of interviews (from 1–7 for faculty, and 1–10 for residents) that particular interview occurred. For example, a faculty member in psychiatry who completed the third faculty interview of the study would be: “F-P-003.”

Note that because the cases were delimited or bound by discipline or residency program, disciplines or programs serve as the units for comparison. Hence, “faculty versus resident” comparisons were not the focus of the study; faculty and residents served as informants to the instrumental case studies where they were collectively analyzed and compared. Yet at times, though not the central focus of the study, faculty versus resident differences were clear enough to warrant reporting.

### Between-case differences in the perceived role of AC and ACEduc

Family medicine and psychiatry perceived AC and ACEduc as a mainstay or crucial part of their work, while surgery equated AC with seeing patients in clinic, secondary to operating. In family medicine, faculty and residents expressed little differentiation of AC.

Obviously, they’re less acutely ill, otherwise, they wouldn’t be presenting in an ambulatory care setting, but from our perspective, this is what we do, and those are the patients that you will be seeing all the time. (F-FM-005)

While AC reportedly encompassed the bulk of psychiatry practice as well, the psychiatry case painted a more distinctive picture of AC and ACEduc compared to family medicine.

[…] Most people live in the community in their homes, contend with their problems most of the week, except maybe during that hour or twenty minutes every couple of weeks when they come in for a session with a practitioner and get some guidance around it. So that’s why I love the educational experience in the ambulatory component because I think it more mirrors what is the reality of people’s lives. (F-P-006)

Surgical residents, in contrast to the other two resident groups, sensed and expressed a deprioritization of ACEduc. They admitted that, while ACEduc may be important, they would still much prefer to operate, and operating and service take priority over ACEduc.

I’m not saying it’s not important, of course it’s important and of course that’s an imperative part of being a surgeon is seeing patients in clinic, it’s just that it doesn’t feel like the most active part. […] absolutely it’s important, but it’s still not as great to most of us as fixing a hernia, being in the operating room, doing what we perceive as the most active part of our training environment. (R-S-002)

Faculty in surgery saw the value of ACEduc more than residents, but corroborated the residents’ perceptions of devaluation, suggesting that surgical and service responsibilities take priority over ACEduc.

And there are service pressures as well and also learners actually want to be in the operating room so if they have a choice. For example, the resident that is with me on Thursday is being asked to cover an operating room at [another hospital] so they do cross over, and somehow that always takes priority, both from the learner but also from a service requirement type of thing. So they will miss the clinic to operate and both are very valuable learning experiences […]. (F-S-001)

At the same time, this may reflect the structure of surgical clinics, with surgeons seeing patients in clinic either before or after operating on them. Most of this care is scheduling or considered preparing for treatment rather than treatment itself. In contrast, with psychiatry and family medicine, the focus in the ambulatory setting is on actually providing treatment.

Residents’ perceptions of surgical staff prioritizing operating time over clinic time seemed to reinforce a less positive view of AC, or clinic, in residents. This privileging of operating room time over any other type of service or learning experience applied not only to the prioritization of patient services over teaching and learning, but also to residents’ stage of training.

So if a senior is in the operating room and you’re on call, or he or she is on call, the junior will still cover the pager while he’s in the operating room, so that [the senior] can get the full operative experience while the junior deals with the other issues. (R-S-001)

### Between-case differences in the structure of ambulatory care education

In family medicine and psychiatry, ACEduc provided a longitudinal and broad patient view, while in surgery, ACEduc occurred episodically. Structure mediated these experiences.

[…] our residents have a practice that they will follow for two years. So, they will see their patients on a regular basis over two years and they will see how a disease process unfolds or if they’re working something up. […]So, there is a really nice piece of longitudinal care and continuity of care that you don’t get in the acute settings. (F-FM-004)

So you’ve got a much richer sense of the context in which people live in, and also a broader sense of the social determinants to their health and well-being, and the importance of things like having educational opportunities, or work opportunities, the importance of recreational opportunities, and the importance of family and friends in people’s lives. You get a much richer sense of that, working with people in an ambulatory care setting, than one usually generally does on the inpatient unit. (F-P-007)

Psychiatry residents suggested the block structure used in their program hampered learning by truncating the ambulatory relationships with patients, remarking that ACEduc as currently configured did not align with actual AC practice, in which a doctor may see a patient for many years.

You don’t really see people over years, which […] you would expect from independent practice, obviously with some other people that you would just see sporadically or just over a short period of time but you really miss that on that long-term component that independent practice brings […] (R-P-009)

The surgical case highlighted the effects of an inconsistent educational structure, with residents reporting a more diffuse exposure to ambulatory patients. In clinic, they would see a patient prior to surgery to make decisions related to surgery. After the surgery, they may or may not see that patient to learn the surgery outcome. In the surgery case, ACEduc meant accumulating fragments of information over time through short interactions. This finding contrasts with family medicine, in which the educational structure allowed for more in-depth study because of the longitudinal relationship with patients.

It’s very unusual in an inpatient setting that you would see a patient in a clinic then operate on them then see them again in the clinic, it’s almost unheard of. (F-S-001)

But, what’s lacking in the ambulatory setting is depth of knowledge. […] in the ambulatory setting, you’re really talking about like 10 to 15-minute interactions. And, if you see a patient in Emerg, again you see that patient for longer but your interaction with your staff about that patient is still relatively brief. So, I feel like in an ambulatory you’re getting sort of snippets of information that accumulate over the number of patients that you see, which is a different type of education, but I don’t know if one is better than the other. (R-S-003)

Some surgical resident participants perceived an expectation to acquire non-medical expert competencies opportunistically, osmotically, and through exposure to role models over time, with little to no formal instruction.

Yeah, so I think on a day-to-day basis the education is focused on our technical skills and a lot of the other stuff is de-emphasized and thought to accumulate passively. […] But, very rarely is somebody actively teaching you about […non-medical expert roles]. And, maybe that’s just because it’s more difficult to teach, and that might also be because of how we’ve learnt, like how they’ve learnt. (R-S-003)

### Across-case hedging on the perceived value of AC and ACEduc

Consistently across all cases and participants, faculty and residents alike, we heard an explicit expression of appreciation for AC. Yet these expressions often rested upon an undercurrent of doubt, as if AC *needed* to be justified. For example:

I think that they recognize the value of ambulatory care even if it’s not what they want to end up doing. I think it’s super useful, right, if you’re seeing someone who … like what a person with bipolar disorder looks like when they’re well, or when they’re kind of just getting manic before they’re sick enough to be admitted, or when they’re just getting depressed before they’re sick enough to be admitted. I think it’s pretty valuable. (R-P-010)

Note, in the excerpt above, this participant adds, “even if it’s not what they want to end up doing” and qualifies AC as “pretty valuable.” These subtle qualifiers, or hedges, were detected across cases and participants, although all participants espoused a value for AC and ACEduc.

### Across-case autonomy through ACEduc

All three cases discussed the autonomous learning and practice afforded by ACEduc. Participants unanimously described ACEduc as a way for residents to expand their skills and independence.

Actually quite a few residents end up identifying that outpatients is harder, not because of the busyness factor but because of the autonomy practice management flexibility pieces, that they actually find that very difficult for them. So I think some residents gravitate towards working on a team, having a structure around them, even if it’s a longer day and some residents like the autonomy self-guided management, flexibility unpredictability of outpatients. (F-P-006)

[…]residents carry their own practices, so we actually have, a grouping of patients should identify their doctor as a resident in our practice. We try very hard to have those patients book primarily with their own resident, unless there’s an urgent issue and they need to be seen on a different day. (F-FM-005)

I found in my education in an ambulatory care setting was more self-directed in the sense that if there was something that I thought or an area of my knowledge that was lacking, it was more something that I had to self-identify and ask a question about. As opposed to an inpatient setting where I found that, or even in the operating room, where somebody is actually identifying holes in your knowledge. (R-S-003)

### Across-case development of competencies through ACEduc

Across all sites and both types of participants, ACEduc reportedly provided excellent opportunities to learn specific roles or competences (e.g., collaboration, advocacy, systems-based practice) in an improved manner.

In the context of mental healthcare, I think one of the things that is unique is that the residents get an opportunity to see clients that are functioning better, and see that there’s an opportunity to see that clients with severe mental illness can be leading more full lives with the support of the mental healthcare services they’re receiving. […] you’ve got a much richer sense of the context in which people live in, and also a broader sense of the social determinants to their health and well-being […] (F-P-007)

[…] you do more of those [intrinsic competency] roles, I guess, in an outpatient setting. […] So, there is, maybe, a little bit more legwork and a little bit more advocacy in communicating and collaboration to do in an outpatient setting, I think, because those resources aren’t at your fingertips. So, I guess it pushes you to go the extra mile with a patient. (R-FM-008)

Notably, the perceived opportunity to learn these non-medical expert roles or competences occurred within a context of challenges. Participants described an uncertain and complex environment of AC, which poses unique difficulties, and valued this challenging environment for its unique learning affordances.

## Discussion

We explored faculty and resident perspectives on ACEduc within and across three residency programs at one medical school. We found points of commonality and differences which present opportunities for targeted educational intervention or further research. While all cases espoused a value for ACEduc, the surgery participants revealed a possible element of discipline-specific hidden curriculum.^49^ Hidden curriculum has been defined as lessons learned, including values and beliefs, in which such lessons were neither explicitly nor intentionally taught by those who control the formal curriculum.^49^ Surgery participants had fewer longitudinal exposures to ACEduc than family medicine and psychiatry, and seemed less enthusiastic about AC, reporting it as less interesting than operating. This finding is reminiscent of Vanstone et al.’s finding amongst neurology and general internal medicine residents, who viewed non-acute patients as a necessary but uninteresting component of their professional practice.^50^ The authors termed this as “resigned professionalism” or “diligent disinterest.”^50^ Surgery participants in our study portrayed a hierarchy in which operating was prioritized over clinic, and service over learning, which interacted to negatively affect residents’ perceptions of the value of ACEduc. According to Vanstone et al., values in the medical learning environment play a strong role with respect to which learning opportunities are recognized by learners.^50^ Lack of formal program learning objectives, limited time, and subtle messages from attending physicians about the value of a learning or patient care context influence trainee uptake of these learning opportunities.^50^ However, given the centrality of operating in surgery, it is not unexpected (and perhaps not unreasonable) that AC and ACEduc have a lesser focus in this specialty as compared with family medicine and psychiatry; this clearly reflects the work of surgery. Furthermore, while protecting surgical residents’ time to attend clinics is an issue, solutions must not only rely on a program emphasizing the importance of the clinic as a learning opportunity. There must also be attention paid to who will do the service work on the wards, emergency room and operating room when residents are in clinic, what the explicit objectives of surgical residency programs are, what the formal schedules and opportunities for learning are, how faculty are role modelling behaviours and values, and what knowledge, skills, and attitudes are assessed. The tension between service and learning was less apparent in family medicine and psychiatry. Surgery participants on the other hand talked more about how time is organized and about positioning residents as human resources, thus affecting the availability and uptake of ACEduc opportunities. This difference across disciplines warrants continued questioning about whether ACEduc should be prioritized across all disciplines, and, if it should, how to provide the cultural and structural conditions for its success. Yet even in family medicine and psychiatry, the explicit espousals of value for ACEduc revealed subtle implicit undertones of skepticism, as if it were bold or daring to express one’s satisfaction with ACEduc over inpatient opportunities.

Also across cases, participants positioned ACEduc as a critical opportunity for non-medical expert competency development, which aligns with prior research.^3^,^21^,^22^ Yet notably, education related to these roles (e.g., collaboration, communication, health advocacy), was generally unstructured, with opportunities varying widely between teachers, and settings. Our participants’ descriptions represent a “random opportunity” approach to learning, as identified in Diachun et al.’s research on geriatric learning on internal medicine clinical teaching units.^51^ In Diachun’s study, despite many opportunities to learn about geriatric care, learners rarely took advantage. The authors concluded that the availability of an opportunity for learning does not guarantee learning will occur.^51^ Our findings of the opportunistic ACEduc approach, particularly in surgery, indicates a need to ask if a curriculum-by-random-opportunity translates into learning of non-medical expert competencies in ACEduc, or, if this is a missed or avoided opportunity, as pointed out by the Diachun et al.^51^ study. Our sense is that, while programs are well intentioned, learning affordances may be at times unavailable, undervalued, *and* unrecognized by residents. Even when they do exist, the pressures of medical culture may not allow learners to fully engage in or embrace such opportunities. For example, high volumes of patients in surgery clinics render non-medical expert roles less important. And, if development and expression of such competencies is of lesser value, this may reinforce high volumes in surgery to the further detriment of competency acquisition. This issue is very complex with many factors at play which extend beyond the scope of our study, but nonetheless warrant further attention (e.g., reasons for acceptance of high volumes in clinic; inadequate number of surgeons to handle the volume, differences in remuneration between surgery time and clinic time, surgeons’ attitudes towards clinic).

Paradoxically, our cases suggested that certain competencies prove exceptionally challenging to learn in ACEduc; yet ACEduc offers advantages to help learn these competencies. For example, participants spoke of challenges related to collaboration in AC. Learning and performing collaboration may be a challenge in AC because access to other health professionals is less readily available than in an inpatient setting. Perhaps collaboration is more complex in AC, because it involves community-based services. Alongside these challenges, participants also identified ACEduc as an ideal setting for self-regulated learning, reflective practice, and non-medical expert competency development. Reasons for this perspective may include the autonomy and realistic experience that ACEduc enables, and the heightened appreciation in AC of patient as “agent” with complex needs and a life within a community, contrasted with the inpatient focus of diagnosing and treating an illness in order to return the patient home. The finding of learners appreciating the unique challenges and opportunities presented by ACEduc aligns well with work on self-regulated learning and desirable difficulty, adaptive expertise, and critical reflective practice, which each embrace challenges as learning opportunities.^55^–^58^ ACEduc may provide research opportunities in these domains.

Translating these findings into possible ACEduc improvements, we consider the educational potential of problem-framing. Cognitive psychology literature on perspective-shifts and scheme, and reflective practice literature on problem-framing, both point to the potential to shape what a learner notices or recalls within their learning experience by (re)focusing their perspective.^52^–^54^ Therefore, as one proposed method to improve ACEduc, we suggest two things. First, we need to determine and agree upon what exactly (e.g., which CanMEDS roles, what knowledge) we believe can be learned most effectively from ACEduc. This study contributes to the knowledge base in this regard. Second, educators and education researchers could then consider more explicitly framing ACEduc as an opportunity to learn those roles and that content from the outset.

### Limitations and conclusions

This collective case study represents a starting point for empirical understandings of AC and ACEduc. Situated in one Canadian medical school, with a limited sample size, we do not aim to generalize our findings beyond these cases (and indeed such generalization would be incongruent with the paradigm of interpretive inquiry). The low return rate on curricular documents pertaining to ACEduc further limited our case study design. Yet a strength of the context-specific nature of our findings within the collective case study design is the insight into mediating contextual factors, or contingencies, in the implementation and experience of ACEduc. These findings generate further questions.

Future research should address whether or how to design ACEduc more purposefully and oriented toward the development of non-medical expertise, when to introduce ACEduc to best afford self-regulated learning and development of expertise, and if and how programs should differentially design ACEduc for different disciplines. How disciplinary culture, program structure, and greater systemic structure influence ACEduc warrant further consideration. In this vein, we suggest investigating how resident and faculty experiences and perspectives of AC *education* relate to discussions of AC itself within the context of health systems reform, lest these efforts work at cross purposes.

In terms of short-term shifts, we suggest explicitly framing and designing ACEduc for learners in a way that aligns with program objectives. If we want learners to embrace ACEduc as a way to learn about advocacy, collaboration, communication, in everyday contexts, then we can and should construct learning objectives, design learning opportunities, and assess our learners accordingly.

## Figures and Tables

**Table 1:** Summary of findings

Between-Case Findings (compare between columns, within rows)	Within-Case Findings FAMILY MEDICINE (look within column)	Within-Case Findings PSYCHIATRY (look within column)	Within-Case Findings SURGERY (look within column)
**Understandings of AC (education)**	Family medicine is AC: continuous, complex, community-based	Psychiatry is largely ambulatory	It means seeing patients in clinic
**The structure of ACEduc**	Longitudinal learning	Longitudinal learning in blocks	Episodic learning
**Overall resident perspectives on AC and ACEduc**	A positive but undifferentiated view of AC and ACEduc	AC and ACEduc are simultaneously and uniquely challenging and interesting	Operating must be prioritized and is more interesting than clinic. AC and ACEduc are valuable depending on one’s stage of learning.
**Overall faculty perspectives on AC and ACEduc**	AC and ACEduc prepare learners for their broad scope of practice	AC and ACEduc pose unique educational challenges and opportunities, and uniquely foster autonomy	Trainees need to see patients to learn non-medical expert competencies. AC teaches decision-making skills that operating does not
**Across-Case Findings:**	ACEduc is a valuable opportunity to more autonomously learn, and to develop non-medical expert competencies (e.g. communication, collaboration, systems-based practice).		
